# Mycobacteria-Specific Mono- and Polyfunctional CD4+ T Cell Profiles in Children With Latent and Active Tuberculosis: A Prospective Proof-of-Concept Study

**DOI:** 10.3389/fimmu.2019.00431

**Published:** 2019-04-05

**Authors:** Marc Tebruegge, Nicole Ritz, Susan Donath, Binita Dutta, Benjamin Forbes, Vanessa Clifford, Christel Zufferey, Robert De Rose, Roy M. Robins-Browne, Willem Hanekom, Stephen M. Graham, Tom Connell, Nigel Curtis

**Affiliations:** ^1^Department of Paediatrics, The University of Melbourne, Parkville, VIC, Australia; ^2^UCL Great Ormond Street Institute of Child Health, University College London, London, United Kingdom; ^3^Academic Unit of Clinical and Experimental Sciences, Faculty of Medicine & Global Health Research Institute, University of Southampton, Southampton, United Kingdom; ^4^Department of Paediatric Infectious Diseases & Immunology, Evelina London Children's Hospital, Guy's and St. Thomas' NHS Foundation Trust, London, United Kingdom; ^5^Infectious Diseases and Pharmacology Unit, University of Basel Children's Hospital, Basel, Switzerland; ^6^Clinical Epidemiology and Biostatistics Unit, Murdoch Children's Research Institute, Parkville, VIC, Australia; ^7^Infectious Diseases Group, Murdoch Children's Research Institute, Parkville, VIC, Australia; ^8^Infectious Diseases Unit, Royal Children's Hospital Melbourne, Parkville, VIC, Australia; ^9^Department of Microbiology and Immunology, Peter Doherty Institute for Infection and Immunity, University of Melbourne, Parkville, VIC, Australia; ^10^Institute of Infectious Diseases and Molecular Medicine and School of Child and Adolescent Health, University of Cape Town, Cape Town, South Africa; ^11^Centre for International Child Health, Parkville, VIC, Australia; ^12^International Child Health Group, Murdoch Children's Research Institute, Parkville, VIC, Australia

**Keywords:** tuberculosis, child, immunoassay, functional profile, T cell, diagnosis

## Abstract

**Background:** Current immune-based TB tests, including the tuberculin skin test (TST) and interferon-gamma release assays (IGRA), have significant limitations, including the inability to distinguish between latent TB infection (LTBI) and active TB. Few biomarkers with the potential to discriminate between these two infection states have been identified.

**Objective:** To determine whether functional profiling of mycobacteria-specific T cells can distinguish between TB-infected and -uninfected children, and simultaneously discriminate between LTBI and active TB.

**Methods:** One hundred and forty-nine children with suspected active TB or risk factors for LTBI were recruited at the Royal Children's Hospital Melbourne. Whole-blood stimulation assays, using ESAT-6, CFP-10, PPD, and heat-killed *M. tuberculosis* as stimulants, were done, followed by intracellular cytokine staining and flow cytometric analysis.

**Results:** Eighty-two participants in the well-defined diagnostic categories ‘uninfected individuals’ (asymptomatic, TST 0 mm / IGRA-; *n* = 61), LTBI (asymptomatic, TST ≥10 mm / IGRA+, normal chest radiograph; *n* = 15), or active TB [microbiologically-confirmed (*n* = 3) or fulfilling stringent criteria (*n* = 3)] were included in the final analysis. The proportions of mycobacteria-specific single-positive TNF-α+ and double-positive IFN-γ+/TNF-α+ CD4+ T cells were significantly higher in participants with active TB than in those with LTBI and uninfected individuals. Additionally, the frequency of IL-17-expressing CD4+ T cells, predominately with single-positive IL-17+ and double-positive IL-2+/IL-17+ phenotypes, was higher in participants with active TB than in the other two groups.

**Conclusions:** The frequencies and functional profiles of mycobacteria-specific CD4+ T cells differ significantly both between TB-infected and TB-uninfected children, and between LTBI and active TB. Although confirmation in further studies will be required, these findings indicate that functional profiling of mycobacteria-specific CD4+ T cells could potentially be exploited for novel immune-based TB assays that enable the distinction between infection states based on a blood sample alone.

## Introduction

Tuberculosis (TB) has recently become the leading infectious cause of death globally (1). The World Health Organization estimates that in 2016 there were 10.4 million new cases of active TB, and 1.6 million TB-related deaths (2). In addition, one-quarter of the global population is thought to be infected with the causative agent of TB, *Mycobacterium tuberculosis* (MTB), and to have asymptomatic latent TB infection (LTBI) (3). Children contribute substantial numbers to this pool of individuals with LTBI, which is fuelling the ongoing TB pandemic (3).

Progress with containing the global pandemic has been hindered substantially by the significant limitations of current diagnostic tools (4). Although considerable advances have recently been made in molecular TB diagnostics (5), and polymerase chain reaction (PCR) based tests are increasingly becoming available in high TB prevalence countries (6), this may not translate into significant improvements in the diagnosis of TB in children, as tests relying on molecular detection of MTB have suboptimal sensitivity in this patient population (7, 8).

Despite intensive research in immunological TB diagnostics, no new immune-based tests have become available for use in routine clinical practice since interferon-gamma (IFN-γ) release assays (IGRAs) were approved in 2002 (4). IGRAs have significant limitations, which include their comparatively high cost, variable reproducibility, largely unexplained discordance with tuberculin skin test (TST) results, and suboptimal sensitivity in both children and adults with active TB (4, 9–13). Furthermore, neither TSTs nor IGRAs can distinguish between LTBI and active TB (4, 14).

Although there is increasing evidence suggesting that these are not distinct entities, but rather extremes on a continuum, the discrimination between LTBI and active TB remains critical from a clinical perspective as their treatment differs (15, 16). A test with the ability to discriminate between LTBI and active TB would be particularly useful in high TB prevalence settings where a large proportion of the population have LTBI, and determining whether a patient with respiratory symptoms and a positive TST or IGRA result has pulmonary TB, or alternatively a respiratory tract infections caused by another pathogen and coincidental LTBI, is often challenging. To date, only a small number of biomarkers that may have the ability to discriminate between LTBI and active TB have been identified (14, 17–20). However, few of these biomarkers have been evaluated in sufficiently large studies, and none have been tested in a routine clinical diagnostic setting.

Advances in flow cytometry over the last decade have enabled increasingly detailed functional profiling of pathogen-specific immune cells. Significant efforts have been made to identify potential correlates of protection against MTB infection, primarily to facilitate the development and evaluation of novel TB vaccines. Mycobacteria-specific polyfunctional T-cells emerged as potential markers of protection, but recent studies have cast doubt on the extent of their role in protective immunity (21). To date, only few studies have explored the use of polyfunctional T-cells in the diagnostic context.

This study aimed to determine the potential of functional profiling of mycobacteria-specific T-cells to distinguish between TB-infected and -uninfected children, and simultaneously discriminate between LTBI and active TB.

## Materials and Methods

### Participants

Children and adolescents up to 18 years of age were recruited at the Royal Children's Hospital Melbourne (RCH) as part of an ongoing project investigating anti-mycobacterial immune responses in children. The inclusion and exclusion criteria for this study are described in detail elsewhere (14). In brief, all children undergoing screening for suspected LTBI or active TB were eligible for inclusion, including the following: (i) children with symptoms and signs suggestive of active TB, (ii) children with known contact with a case of active TB, (iii) children who had recently migrated from countries with a high TB prevalence (incidence ≥ 40 TB cases/100,000 population). Children with known immunodeficiency and those receiving immunosuppressive treatment were excluded from participation. Potential participants who had a TST in the previous 6–52 weeks were excluded from participation, as at commencement of the study there were some data suggesting that a TST done 6 weeks prior to an IGRA may result in boosting, thereby causing false-positive IGRA results. We have subsequently shown that this is not the case (22).

Informed consent was obtained from each child's parent and/or guardian. A standardized data collection sheet was used to record demographics, history, and clinical findings. A chest x-ray was done in all cases with positive TST or IGRA results. Histological, conventional, and molecular microbiological tests were done in all children with suspected active TB as clinically indicated.

### Diagnostic Tests

All participants had a TST performed by intradermal injection of 0.1 ml Tubersol (Sanofi Pasteur; Toronto, Canada; bioequivalent to 5 Tuberculin Units PPD-S), and the resulting induration was recorded after 48–72 h. In addition, blood was obtained for the QuantiFERON-TB Gold In-Tube (QFT-GIT; Cellestis/Qiagen; Carnegie, Australia) assay, which was processed and interpreted at the Victorian Infectious Diseases Reference Laboratories (VIDRL) according to manufacturer's instructions. An additional 10 mL of blood was collected in heparinized tubes for whole blood assays. In addition to standard microcopy and culture, polymerase chain reaction (PCR) testing for MTB, done with a Taqman real-time PCR (Applied Biosystems; Waltham, MA) targeting the insertion sequence IS6110, was done at VIDRL, using previously described methods (23).

### Categorization of Participants and Definitions

Participants were classified into three well-defined categories according to their clinical features, TST, IGRA, and microbiological results: (i) uninfected individuals, (ii) individuals with LTBI, and (iii) individuals with active TB. Uninfected individuals were defined as asymptomatic participants without any palpable TST induration (i.e., TST result of 0 mm), and a negative QFT-GIT result. LTBI was defined as asymptomatic individuals with a positive TST result (≥10 mm), a positive QFT-GIT result and an unremarkable chest radiograph. Active TB was defined as either (i) microbiological confirmation of infection with MTB by culture or PCR, or (ii) a symptomatic individual fulfilling at least two of the following three criteria in conjunction with response to treatment with anti-tuberculous therapy: (a) symptoms and signs consistent with active TB (chronic cough, persistent fever, night sweats, unexplained weight loss), (b) radiological findings suggestive of active TB, (c) presence of risk factors for TB infection (known TB contact, birth or previous residence in a high TB prevalence country). These stringent criteria exceed those proposed by the American Thoracic Society and the Centers for Disease Control and Prevention (24). Participants who did not fall into any of these three distinct diagnostic categories, including asymptomatic individuals with a TST induration of 1–9 mm, were excluded from further analyses to ensure unambiguous diagnostic groups.

### Whole Blood Assays

Whole blood was incubated with ESAT-6, CFP-10 (each at a concentration of 10 μg/ml; JPT Peptide Technologies, Berlin, Germany), PPD (20 μg/ml; RT50; Statens Serum Institut, Copenhagen, Denmark), heat-killed MTB H37Rv (MTBk; 1.6 × 10^6^ CFU/ml), staphylococcal enterotoxin B (5 μg/ml; positive control; Sigma-Aldrich, St. Louis, MO), or without stimulant (negative control) in the presence of co-stimulatory antibodies, anti-CD28 and anti-CD49d (each 1 μg/ml; BD Biosciences, San Jose, CA). Following incubation at 37°C for 20 to 24 h, brefeldin A (10 μg/ml; Sigma-Aldrich, St. Louis, MO) was added, and samples were incubated for a further 5 h. Following addition of EDTA (2 mM; Sigma-Aldrich) samples were transferred into FACS lysing solution (BD Biosciences), and then cryopreserved at −80°C for batched analysis.

### Cell Staining and Flow Cytometric Analysis

**Figure 1:**
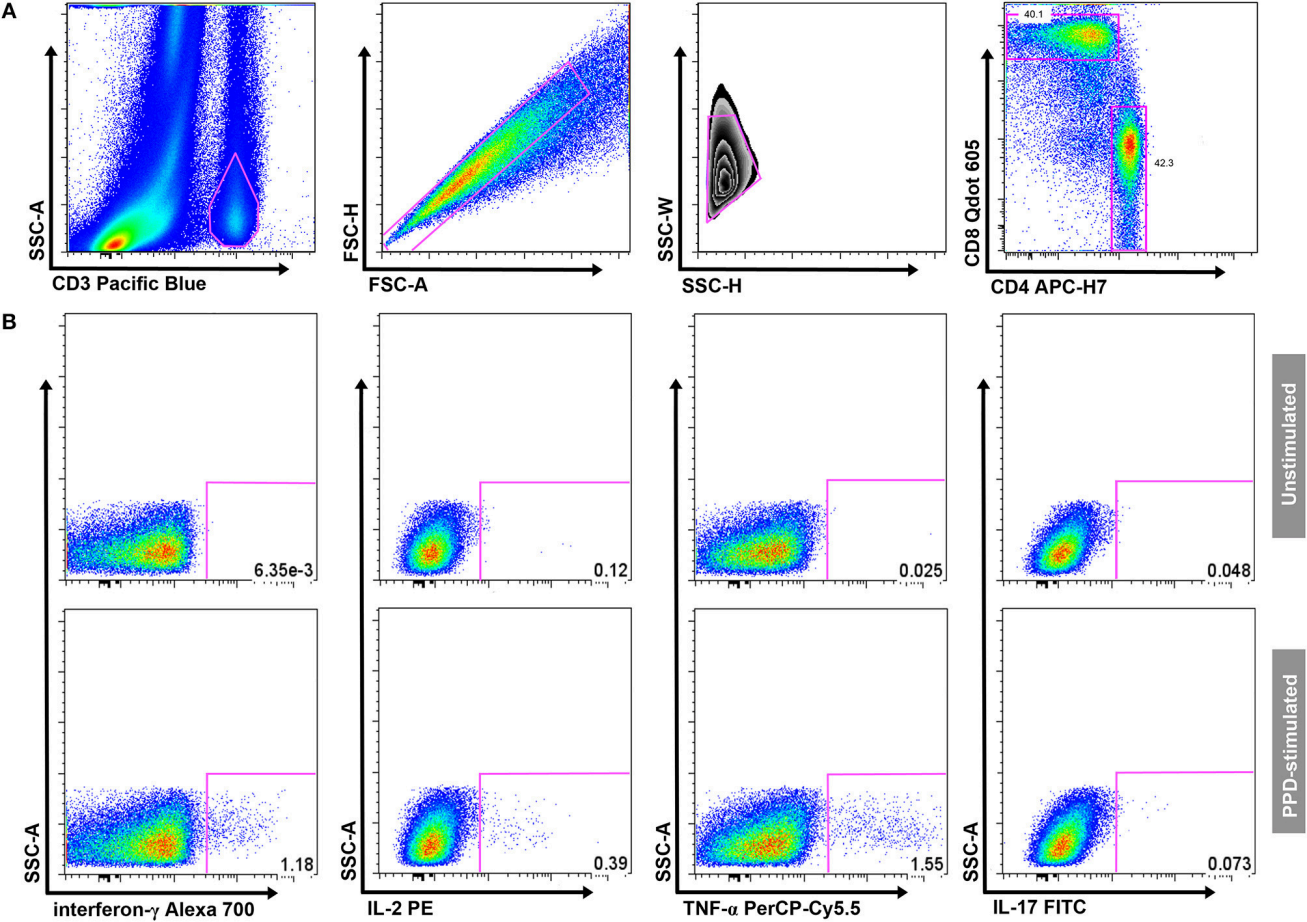
Gating strategy. **(A)** Gating of CD3+ T cells, aggregate exclusion, followed by gating of single CD4+ and CD8+ T cells. **(B)** Threshold gates for CD4+ T cells producing IFN-γ, IL-2, TNF-α, or IL-17; a Boolean gating strategy was used to determine the proportion of polyfunctional T-cells. Gates for cytokine-producing CD4+ T cells were set on the unstimulated sample (upper panel) and transposed onto the antigen-stimulated samples (in this example PPD; lower panel) in each individual participant.

Samples were thawed and permeabilized with Perm2 Solution (BD Biosciences) for 10 min. Cells were then washed with 1xPBS/0.5%BSA/0.1%NaN3 staining buffer before incubation with fluorochrome-conjugated antibodies at room temperature for 30 min. The following antibodies were used: anti-CD3 Pacific Blue (UCHT1), anti-CD4 APC-H7 (RPA-T4), anti-CD8 Qdot 605 (3B5), anti-IFN-γ Alexa 700 (B27), anti-IL-2 PE (MQ1-17H12), anti-TNF-α PerCP-Cy5.5 (MAb11), anti-IL-17 FITC (BL168) [all BD Biosciences, except Qdot-605 (Life Technologies, Carlsbad, CA) and FITC (BioLegend, San Diego, CA)]. Cells were analyzed on a LSRII flow cytometer (BD Biosciences). Cytometer Setup and Tracking beads (BD Biosciences) were run prior to acquisition to optimize instrument settings. Automated compensations were calculated with FACSDiva software (BD Biosciences) using stained CompBeads (Pacific-Blue, APC-H7, Qdot-605, PerCP-Cy5.5), stained CompBeads Plus (Alexa-700), and Calibrite (PE, FITC) beads (all BD Biosciences). Analysis was performed using FlowJo software (version 8.8.6; TreeStar Inc., Ashland, OR). A hierarchical gating strategy was used to select single-cell CD4 and CD8 T-cell populations. Gates for cytokine expression in samples stimulated with mycobacterial antigens were set using the unstimulated control sample (Figure 1). A Boolean combination was used to determine polyfunctional T-cells producing two or more cytokines.

### Statistical Analysis and Reporting

Statistical analyses were done with Stata (Version 12; StataCorp, College Station, TX) and Prism (Version 5; Graph Pad Software Inc., La Jolla, CA). Data were background-corrected prior to analysis by subtracting the proportion of cytokine-producing cells detected in unstimulated (nil control) samples from the proportion detected in antigen-stimulated samples. Comparisons of continuous variables between multiple groups were done using non-parametric Kruskal–Wallis tests. In instances where the Kruskal–Wallis *p*-value was < 0.1, indicating a potential difference between the groups, additional analyses using two sided Mann–Whitney *U*-tests for two group comparisons were done. Mann–Whitney *U p*-values < 0.05 were considered statistically significant. All figures were constructed with Prism.

## Results

**Figure 2:**
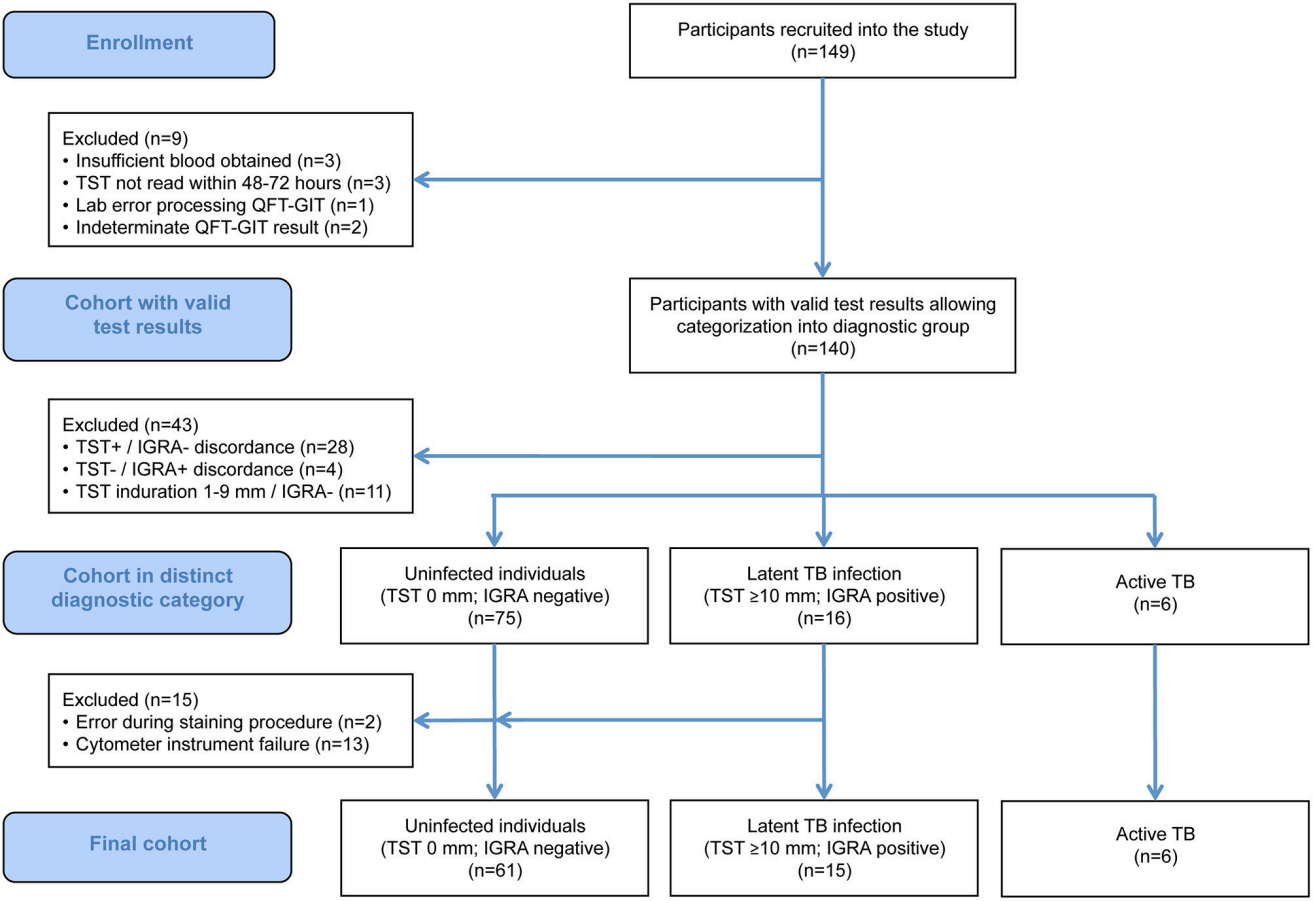
STARD flow chart showing the composition of the initial and the final study population. IGRA, interferon-γ release assay; QFT-GIT, QuantiFERON-TB Gold In-Tube assay; TB, tuberculosis; TST, tuberculin skin test.

A total of 149 participants were recruited into the study. Of these, 97 fulfilled the study criteria for the diagnostic categories uninfected (*n* = 75), LTBI (*n* = 16), and active TB (*n* = 6). Of these, 14 participants in the uninfected and one in the LTBI group had to be excluded due to technical issues during sample analysis (mainly instrument failure; Figure 2). Therefore, a total of 82 participants, entailing 82 unstimulated and 328 antigen-stimulated samples, were included in the final analysis.

The baseline characteristics of the study population are summarized in Table 1. The six participants with active TB comprised three patients with microbiologically-confirmed TB (intrathoracic TB, *n* = 1; lymph node TB, *n* = 2) and three patients who fulfilled the study criteria for active TB without microbiological confirmation (pulmonary TB, *n* = 2; spinal TB, *n* = 1). All six had positive TST and QFT-GIT results, and had resolution of symptoms with anti-tuberculous treatment. Further details on these patients can be found in our previous publication (14).

**Table 1:** Baseline characteristics of the study population.

	**Uninfected**	**LTBI**	**Active TB**
	**(*n* = 61)**	**(*n* = 15)**	**(*n* = 6)**
Median (IQR) age, years	5.7	11.7	15.0
	(2.8–10.8)	(5.6–14.4)	(12.1–16.2)
**Ethnic origin, no. (%)**			
Africa	27 (44.3)	10 (66.7)	5 (83.3)
Asia	19 (31.1)	4 (26.7)	1 (16.7)
Middle East	4 (6.6)	0	0
Australia/New Zealand	11 (18.0)	1 (6.7)	0
Migration background ^*^, no. (%)	28 (45.9)	14 (93.3)	6 (100)
Median (IQR) duration of residence in Australia (migrants only ^*^), months	7.0 (2.3–36.0)	12.5 (4.0–37.3)	21.0 (3.0–69.0)
**BCG vaccination history, no. (%)**			
Yes	21 (34.4)	13 (86.7)	5 (83.3)
No	37 (60.6)	1 (6.7)	1 (16.7)
Unknown	3 (4.9)	1 (6.7)	0
**BCG vaccination scar, no. (%)**			
Yes	20 (32.8)	11 (73.3)	5 (83.3)
No	41 (67.2)	4 (26.7)	1 (16.7)
**Known TB contact, no. (%)**			
Yes	42 (68.9)	7 (46.7)	1 (16.7)
No	19 (31.1)	8 (53.3)	5 (83.3)
**Type of TB contact, no. (%)**			
Parent	10 (16.4)	3 (20.0)	1 (16.7)
Other household member	20 (32.8)	3 (20.0)	0
Other contact	12 (19.7)	1 (6.7)	0
No known contact	19 (31.1)	8 (53.3)	5 (83.3)

*BCG, Bacillus Calmette-Guérin; IQR, interquartile range; LTBI, latent tuberculosis infection; TB, tuberculosis*.

* *Excludes migrants from New Zealand*.

### Total Mycobacteria-Specific, Cytokine-Producing CD4+ T-Cells

**Figure 3:**
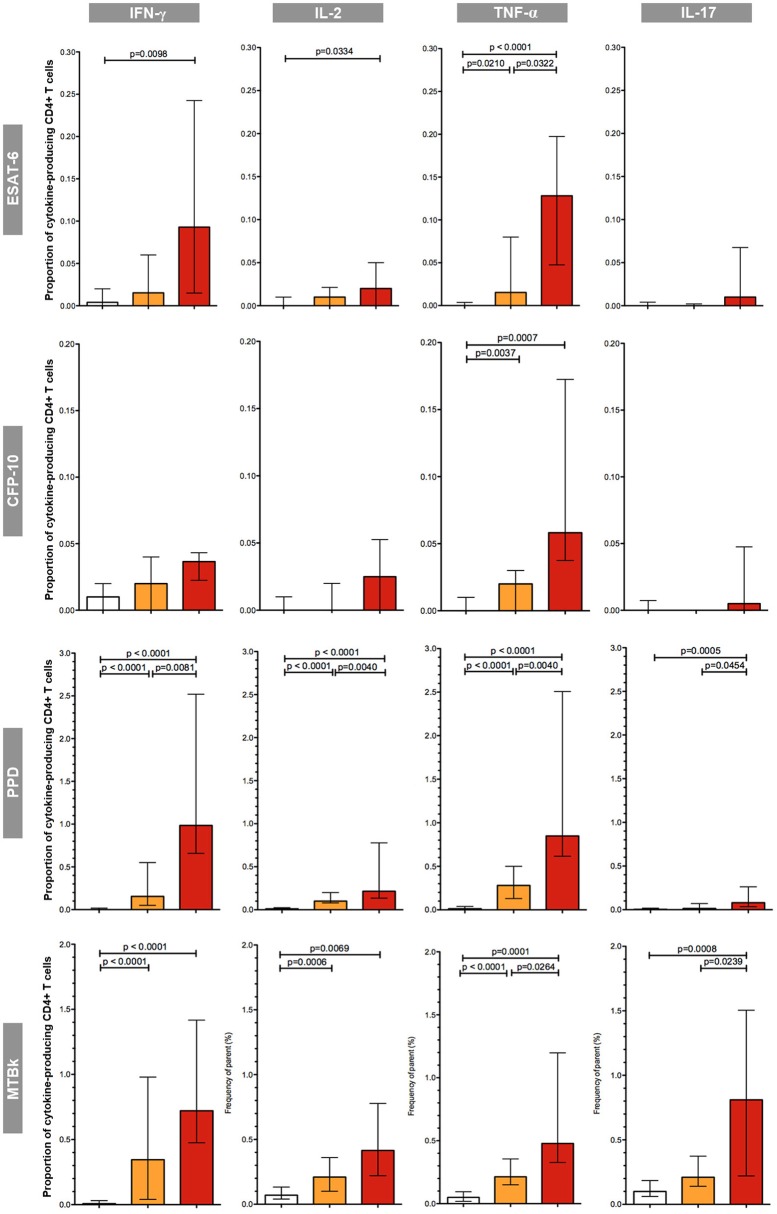
Proportions of mycobacteria-specific, cytokine-producing CD4+ T cells induced by antigenic stimulation in the three diagnostic groups. The different groups are indicated by the color: uninfected (white boxes), latent tuberculosis infection (orange boxes), active tuberculosis (red boxes). The respective antigenic stimulant is indicated on the left (note scales vary between stimulants), and the cytokines on the top of the figure. The boxes represent the medians; the whiskers indicate the IQR. All data shown are background-corrected. *P*-values were calculated with Mann-Whitney *U*-tests; all significant *p*-values are shown in the figure.

First, the proportion of mycobacteria-specific CD4+ T-cells producing IFN-γ, IL-2, TNF-α, or IL-17 in antigen-stimulated samples was determined (Figure 3).

In the group of uninfected individuals, the median proportions of cytokine-producing CD4+ T-cells were universally low; proportions were consistently below 0.1%, irrespective of the stimulant used. Overall, in both the LTBI and the active TB group the proportions of CD4+ T-cells producing cytokines in response to stimulation with mycobacterial antigens were considerably greater in PPD and MTBk stimulated samples compared to samples stimulated with the MTB-specific peptide antigens ESAT-6 and CFP-10. ESAT-6 induced greater proportions of cytokine-producing CD4+ T-cells than CFP-10. Stimulation with ESAT-6, CFP-10, and PPD induced only small proportions of IL-17+ CD4+ T-cells, while stimulation with MTBk resulted in substantially higher proportions of these cells.

Irrespective of the stimulant used, the proportions of IFN-γ+, IL-2+, TNF-α+, and IL-17+ CD4+ T-cells were highest in the group with active TB. In ESAT-6, PPD, and MTBk stimulated samples, the median proportions of TNF-α+ CD4+ T-cells were significantly higher in participants with active TB than in those with LTBI (0.13 vs. 0.02%, *p* = 0.032; 0.85 vs. 0.28%, *p* = 0.004; 0.48 vs. 0.21%, *p* = 0.026, respectively). In PPD and MTBk stimulated samples the median proportions of IL-17+ CD4+ T-cells were significantly higher in the group with active TB than in the group with LTBI (0.08 vs. 0.01%, *p* = 0.045; 0.81 vs. 0.21%, *p* = 0.024, respectively).

### Mycobacteria-Specific Mono- and Polyfunctional CD4+ T-Cells

**Figure 4:**
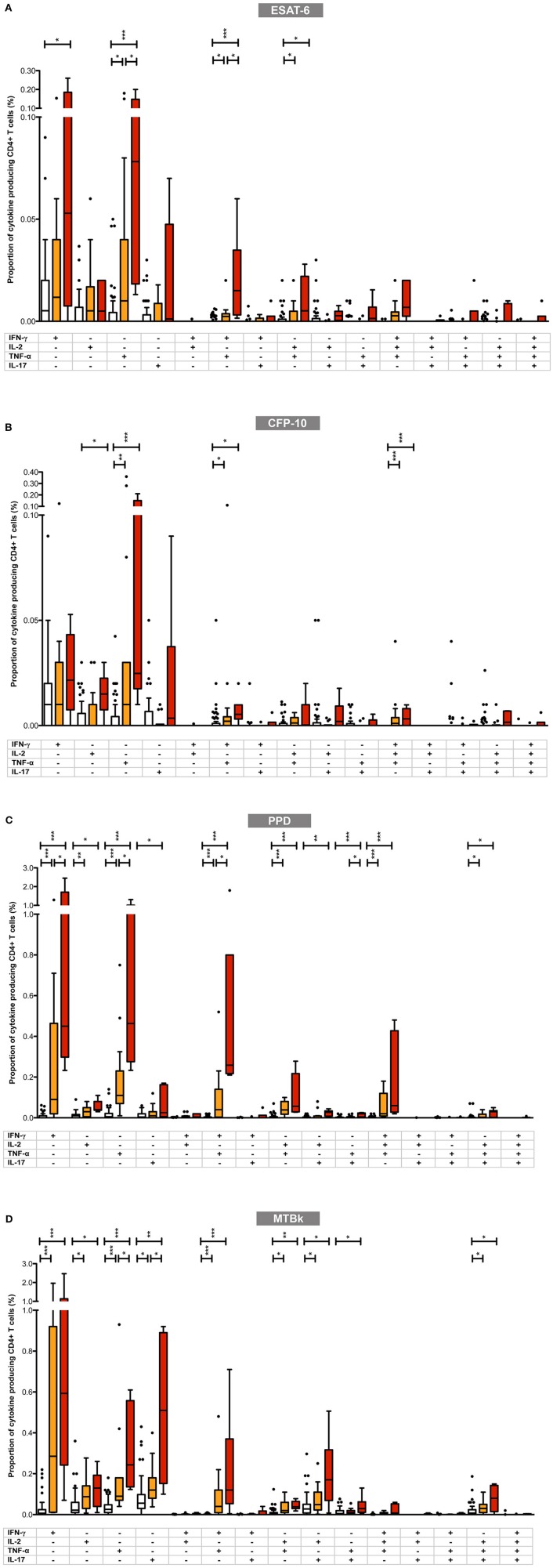
Proportions of mycobacteria-specific mono- and polyfunctional CD4+ T cells in the three diagnostic groups in **(A)** ESAT-6, **(B)** CFP-10, **(C)** PPD, and **(D)** MTBk stimulated samples. Box plot with Tukey whiskers; the boxes represent the interquartile range and the horizontal lines indicate the medians. The different groups are indicated by the color: uninfected (white boxes), latent tuberculosis infection (orange boxes), active tuberculosis (red boxes). All possible combinations of cytokine expression are shown on the x-axis (single cytokine producing CD4+ T cells toward the left; polyfunctional CD4+ T cells toward the right). All data shown are background-corrected. Statistically significant differences between the three diagnostic groups are indicated by star symbols: ^*^p < 0.05; ^**^p < 0.001; ^***^p < 0.0001.

Next we determined whether the pattern and proportions of single-cytokine producing and polyfunctional CD4+ T-cells differed between the three diagnostic groups. The results of these analyses are summarized in Figures 4A–D and in Supplementary Table 1.

Overall, in participants with LTBI and active TB, the proportions of mycobacteria-specific monofunctional, single-cytokine producing CD4+ T-cells were higher than those of double-, triple- and quadruple-positive polyfunctional CD4+ T-cells. With ESAT-6, CFP-10, and PPD stimulation, the highest proportions of mycobacteria-specific CD4+ T-cells comprised cells with the following functional profiles: IFN-γ+ only, TNF-α+ only, IFN-γ+/TNF-α+, IL-2+/TNF-α+, and IFN-γ+/IL-2+/TNF-α+. This pattern differed from that observed in MTBk stimulated samples, where the following functional profiles predominated: IFN-γ+ only, TNF-α+ only, IL-17+ only, IFN-γ+/TNF-α+, IL-2+/IL-17+, IL-2+/TNF-α+/IL-17+.

Although there was a general tendency for the proportions of mono- and polyfunctional CD4+ T-cells to be higher in the active TB group than in the LTBI group, only few comparisons between these two groups reached statistical significance. Irrespective of the stimulant used, the largest differences between the two groups were consistently observed in single-positive TNF-α+ CD4+ T-cells (statistically significant in ESAT-6, PPD, and MTBk stimulated samples) and double-positive IFN-γ+/TNF-α+ CD4+ T-cells (statistically significant in ESAT-6 and PPD stimulated samples). In MTBk stimulated samples the proportion of single-positive IL-17+ CD4+ T-cells was significantly higher in the active TB group than in the LTBI group, contrasting with the observations in ESAT-6, CFP-10 and PPD stimulated samples.

## Discussion

Our data indicate that analysis of mycobacteria-specific CD4+ T-cell responses can distinguish between TB-infected and TB-uninfected children. In addition, we found that the proportions of certain mycobacteria-specific mono- and polyfunctional CD4+ T-cells differed significantly between individuals with LTBI and patients with active TB. The most marked differences between these two groups were observed in single-positive TNF-α+ CD4+ T-cells and double-positive IFN-γ+/TNF-α+ CD4+ T-cells, a finding that could be exploited for diagnostic purposes.

The observation that CD4+ T-cells expressing TNF-α are the hallmark of active TB in children is consistent with recent observations reported from a study in adults, which included a discovery cohort comprising 48 individuals with LTBI and eight cases with active TB (18). Using a similar approach to functional profiling of mycobacteria-specific T-cells with intracellular cytokine staining, the investigators found that adults with active TB had significantly higher proportions of single-positive TNF-α+ CD4+ T-cells than those with LTBI. However, in contrast to our study that used whole blood assays, peripheral blood mononuclear cells (PBMCs) were used, and the functional analyses were restricted to IFN-γ, IL-2, and TNF-α expression. In contrast to our findings, the investigators reported that polyfunctional CD4+ T-cells expressing all three cytokines predominated in adults with LTBI. Irrespective of the antigenic stimulant used, we did not observe any significant differences between children with LTBI and those with active TB in relation to mycobacteria-specific IFN-γ+/IL-2+/TNF-α+ CD4+ T-cells, and in both groups the proportions of triple-positive polyfunctional CD4+ T-cells were generally considerably smaller than the proportions of single-positive CD4+ T-cells. Our findings are consistent with a recent study that included 15 adults with LTBI but no cases with active TB, which also found that the frequency of mycobacteria-specific single-positive TNF-α+ CD4+ T-cells in this patient group was far greater than the frequency of triple-positive cells (25). However, all subjects in that study were HIV-infected and the analyses primarily focused on memory T-cells. Another study in adults with LTBI, which used MTB DosR antigens regulated by Rv3313c as stimulants, also found that mycobacteria-specific single-cytokine producing T-cells predominated in individuals with LTBI, rather than double-positive and triple-positive IFN-γ+/IL-2+/TNF-α+ T-cells (26). Ultimately, additional studies applying identical methods to samples from both children and adults will be required to determine whether there are truly age-related differences in the functional profiles of mycobacteria-specific T-cells, or whether these contrasting observations are the result of methodological differences.

In addition to single-positive TNF-α+ CD4+ T-cells, we found that the frequency of mycobacteria-specific polyfunctional IFN-γ+/TNF-α+ CD4+ T-cells was significantly greater in children with active TB than in those with LTBI. This observation is consistent with a study by Sutherland and colleagues, which compared functional T-cell profiles between adults with active TB and asymptomatic household contacts (27). Unfortunately, TST-negative and TST-positive (i.e., individuals likely to have LTBI) household contacts were combined into one group in some of the analyses, precluding direct comparisons. Nevertheless, using an ESAT-6/CFP-10 fusion protein and PPD as stimulants, the investigators detected significantly greater proportions of double-positive IFN-γ+/TNF-α+ CD4+ T-cells in the active TB group than in the comparator group. A relatively small, more recent study that included 13 adults with active TB and 21 with LTBI (defined as asymptomatic individuals with positive ELISpot responses and no evidence of active TB), also made similar observations as our study (28). Those with active TB were found to have significantly higher proportions of PPD-specific single-positive TNF-α+ and double-positive IFN-γ+/TNF-α+ CD4+ T-cells than individuals with LTBI. In accordance with our findings, no difference in the proportions of triple-positive IFN-γ+/IL-2+/TNF-α+ T-cells was observed between the two groups.

There has long been strong evidence that TNF-α is a key cytokine in anti-mycobacterial immune responses in both animals and in humans (29). The observation that patients treated with monoclonal anti-TNF-α antibodies for chronic inflammatory conditions are at significantly increased risk of progression from LTBI to active TB has added to the existing evidence (30). Recent data suggest that TNF-α may also be a key cytokine for the distinction between LTBI and active TB. We have recently reported that TNF-α concentrations in supernatants harvested from whole blood stimulation assays were significantly higher in children with active TB than in children with LTBI in a cohort that largely overlapped with the cohort described in the present report (14). Furthermore, by combining TNF-α responses with IL-10 and IL-1ra responses, patients could be accurately classified into one of these two infection states. Similar observations regarding mycobacteria-specific TNF-α responses being of greater magnitude in patients with active TB than in individuals with LTBI have also been reported by adult studies (20, 31, 32).

IGRAs, which are currently the only *in vitro* immunodiagnostic test for TB approved for clinical use, are based solely on the detection of IFN-γ responses following stimulation with mycobacterial peptides. A large number of studies provide evidence that IFN-γ responses do not differ between individuals with LTBI and patients with active TB (14, 20, 33). Therefore, neither IGRAs nor other, non-commercial IFN-γ-based assays can be used to discriminate between those two infection states. Inclusion of TNF-α responses, potentially in combination with other discriminatory biomarkers, into future immune-based assays for TB is likely to increase assay sensitivity and simultaneously provide useful additional information about the probable infection state to help guide management. Other biomarkers with the potential to distinguish between TB infection states, including CD38, Ki67, and HLA-DR (34–36), also warrant further investigation.

A further intriguing finding is the observation that mycobacteria-specific IL-17 responses appear to depend on the antigen used for stimulation. Although some IL-17 producing CD4+ T-cells were detected in active TB patients following stimulation with the MTB-specific peptide antigens ESAT-6 and CFP-10, as well as PPD, the proportions of these cells were several-fold greater when heat-killed MTB was used as the stimulant. This raises the question whether certain antigens contained in heat-killed MTB, but not in PPD, such as lipid antigens, are particularly potent inducers of IL-17 responses. Notably, recent data show that mycolic acids, lipid antigens contained in the cell wall of MTB, are strong inducers of IL-17 in CD1b-restricted T cells (37). Also, in both PPD and heat-killed MTB stimulated samples, the proportions of mycobacteria-specific IL-17+ CD4+ T-cells were significantly greater in patients with active TB than in individuals with LTBI. The majority of mycobacteria-specific CD4+ T-cells expressing IL-17 had single-positive IL-17+ and double-positive IL-2+/IL-17+ phenotypes. Our observations are in accordance with previous reports in adults. One study that included adult patients with active TB and healthy BCG-vaccinated donors, which analyzed PBMCs stimulated with MTB extracts, found that the proportion of IL-17+ T-cells was substantially higher in the former group (38). In another study, which included active TB patients and adults with LTBI (based on positive TST results) and analyzed PBMCs after stimulation with different MTB strains, found that active TB cases had significantly higher proportions of IL-17+ CD4+ T-cells (39). Interestingly, recent data in adults also suggest that IL-17 expression in T-cells may correlate with disease severity in active TB (40). The importance of Th17 cells, which characteristically produce IL-17, IL-21, and IL-22, in the anti-mycobacterial immune response has been discovered a decade ago (41, 42). Current concepts suggest that the main role of IL-17 lies in the early stages of MTB infection, and specifically in promoting neutrophil recruitment and survival, thereby aiding granuloma formation and containment of the pathogen.

The main limitation of our study is the inclusion of a limited number of patients with active TB, a limitation shared by many other studies in this area that have included a similar number of cases (17, 18, 33). However, despite this, we detected a number of highly significant differences between TB-infected and TB-uninfected individuals, and between individuals with LTBI and patients with active TB. A further limitation is the absence of an additional control group of children who had symptoms compatible with TB but with an alternative subsequent diagnosis. An important strength of this study is the use of unambiguous diagnostic groups in the analyses. This approach led to the exclusion of a large proportion of the initial study population from the final cohort, but considerably strengthens our data. Many previous immunodiagnostic studies have included participants with uncertain TB infection status (e.g., solely based on TST results) or uncertain active TB cases (e.g., “possible” and “probable TB” cases based exclusively on clinical features), and therefore have an inherent risk of data contamination confounding the analyses.

In conclusion, we found that profiling of mycobacteria-specific CD4+ T-cell responses has the potential to discriminate between TB-infected and TB-uninfected children, and simultaneously between children with LTBI and those with active TB. Mycobacteria-specific single-positive TNF-α+ and double-positive IFN-γ+/TNF-α+ CD4+ T-cells are the hallmark of active TB in children. In addition, compared with children with LTBI, children with active TB have significantly higher proportions of IL-17 expressing mycobacteria-specific CD4+ T-cells, mainly with single-positive IL-17+ and double-positive IL-2+/IL-17+ phenotypes. Further studies will be required to determine whether these findings can be translated into novel immune-based diagnostic assays that allow the distinction between infection states in the clinical setting.

## Ethics Statement

The study was approved by the RCH Human Research Ethics Committee (HREC 29040A).

## Author Contributions

MT, NR, SD, BD, RD, RR-B, SG, TC, and NC conception and design. MT, BD, and BF acquisition of data. MT, NR, SD, BD, BF, VC, CZ, RD, RR-B, WH, SG, TC, and NC analysis and interpretation of data. MT and NC drafting the manuscript. NR, SD, BD, BF, VC, CZ, RD, RR-B, WH, SG, and TC revising the manuscript for important intellectual content.

### Conflict of Interest Statement

The authors declare that the research was conducted in the absence of any commercial or financial relationships that could be construed as a potential conflict of interest.
