# Supplementary material for: Mycobacteria-Specific Mono- and Polyfunctional CD4+ T Cell Profiles in Children With Latent and Active Tuberculosis: A Prospective Proof-of-Concept Study
Source: Front Immunol. 2019 Apr 5;10:431. doi: 10.3389/fimmu.2019.00431 (PMC6459895; doi:10.3389/fimmu.2019.00431)
Supplement: Supplementary file 1 [file Table_1.pdf]

Supplementary Table 1. Median proportions and interquartile ranges of mycobacteria-specific mono- and polyfunctional CD4+ T cells in the three diagnostic groups according to antigenic stimulant. The values shown in the last three columns were calculated by Mann Whitney *U* tests; statistically significant Mann Whitney *U* p-values ($p < 0.05$) are highlighted in red.

Stimulant	Cytokine(s) produced	Median percentage (IQR)			Kruskal Wallis p-value	Un-infected vs. LTBI	Un-infected vs. Active TB	LTBI vs. Active TB
		Un-infected	LTBI	Active TB				
ESAT-6	IFN-γ (single)	0.005 (0.001-0.020)	0.012 (0.000-0.040)	0.053 (0.080-0.185)	0.0867	0.2870	0.0163	0.1317
	IL-2 (single)	0.000 (0.000-0.007)	0.005 (0.000-0.017)	0.005 (0.000-0.020)	0.3409	-	-	-
	TNF-α (single)	0.000 (0.000-0.004)	0.010 (0.000-0.040)	0.078 (0.018-0.147)	< 0.0001	0.0077	< 0.0001	0.0377
	IL-17 (single)	0.000 (0.000-0.003)	0.000 (0.000-0.009)	0.001 (0.000-0.048)	0.2764	-	-	-
	IFN-γ/IL-2	0.000 (0.000-0.000)	0.000 (0.000-0.000)	0.000 (0.000-0.000)	§	-	-	-
	IFN-γ/TNF-α	0.000 (0.000-0.000)	0.002 (0.000-0.004)	0.015 (0.003-0.035)	< 0.0001	0.0020	< 0.0001	0.0146
	IFN-γ/IL-17	0.000 (0.000-0.000)	0.000 (0.000-0.002)	0.000 (0.000-0.002)	§	-	-	-
	IL-2/TNF-α	0.000 (0.000-0.001)	0.000 (0.000-0.005)	0.005 (0.000-0.022)	0.0491	0.0379	0.0281	0.2916
	IL-2/IL-17	0.000 (0.000-0.001)	0.000 (0.000-0.000)	0.002 (0.000-0.009)	§	-	-	-
	TNF-α/IL-17	0.000 (0.000-0.000)	0.000 (0.000-0.000)	0.001 (0.000-0.007)	§	-	-	-
	IFN-γ/IL-2/TNF-α	0.000 (0.000-0.000)	0.003 (0.000-0.005)	0.007 (0.002-0.020)	§	-	-	-
	IFN-γ/IL-2/IL-17	0.000 (0.000-0.000)	0.000 (0.000-0.000)	0.000 (0.000-0.001)	§	-	-	-
	IFN-γ/TNF-α/IL-17	0.000 (0.000-0.000)	0.000 (0.000-0.000)	0.000 (0.000-0.005)	§	-	-	-
	IL-2/TNF-α/IL-17	0.000 (0.000-0.000)	0.000 (0.000-0.000)	0.000 (0.000-0.009)	§	-	-	-
	IFN-γ/IL-2/TNF-α/IL-17	0.000 (0.000-0.000)	0.000 (0.000-0.000)	0.000 (0.000-0.002)	§	-	-	-
CFP-10	IFN-γ (single)	0.010 (0.000-0.020)	0.010 (0.000-0.030)	0.022 (0.007-0.043)	0.3370	-	-	-
	IL-2 (single)	0.000 (0.000-0.006)	0.000 (0.000-0.020)	0.015 (0.007-0.022)	0.0446	0.3986	0.0065	0.0962
	TNF-α (single)	0.000 (0.000-0.006)	0.010 (0.000-0.030)	0.025 (0.018-0.150)	< 0.0001	0.0008	< 0.0001	0.1477
	IL-17 (single)	0.000 (0.000-0.007)	0.000 (0.000-0.001)	0.003 (0.000-0.038)	0.2434	-	-	-
	IFN-γ/IL-2	0.000 (0.000-0.000)	0.000 (0.000-0.000)	0.000 (0.000-0.000)	§	-	-	-
	IFN-γ/TNF-α	0.000 (0.000-0.001)	0.002 (0.000-0.004)	0.005 (0.003-0.010)	0.0029	0.0381	0.0022	0.1384
	IFN-γ/IL-17	0.000 (0.000-0.000)	0.000 (0.000-0.000)	0.000 (0.000-0.002)	§	-	-	-
	IL-2/TNF-α	0.000 (0.000-0.001)	0.001 (0.000-0.004)	0.000 (0.000-0.010)	0.2263	-	-	-
	IL-2/IL-17	0.000 (0.000-0.001)	0.000 (0.000-0.000)	0.002 (0.000-0.009)	§	-	-	-
	TNF-α/IL-17	0.000 (0.000-0.001)	0.000 (0.000-0.000)	0.000 (0.000-0.003)	§	-	-	-
	IFN-γ/IL-2/TNF-α	0.000 (0.000-0.000)	0.001 (0.000-0.004)	0.003 (0.000-0.008)	< 0.0001	< 0.0001	< 0.0001	0.6028
	IFN-γ/IL-2/IL-17	0.000 (0.000-0.000)	0.000 (0.000-0.000)	0.000 (0.000-0.001)	§	-	-	-
	IFN-γ/TNF-α/IL-17	0.000 (0.000-0.000)	0.000 (0.000-0.000)	0.000 (0.000-0.001)	§	-	-	-
	IL-2/TNF-α/IL-17	0.000 (0.000-0.000)	0.000 (0.000-0.000)	0.002 (0.000-0.007)	§	-	-	-
	IFN-γ/IL-2/TNF-α/IL-17	0.000 (0.000-0.000)	0.000 (0.000-0.000)	0.000 (0.000-0.000)	§	-	-	-

§ Insufficient number of unique values to calculate valid p-value.

Stimulant	Cytokine(s) produced	Median percentage (IQR)			Kruskal Wallis p-value	Un-infected vs. LTBI	Un-infected vs. Active TB	LTBI vs. Active TB
		Un-infected	LTBI	Active TB				
PPD	IFN-γ (single)	0.000 (0.000-0.010)	0.090 (0.020-0.464)	0.450 (0.298-1.702)	< 0.0001	< 0.0001	< 0.0001	0.0239
	IL-2 (single)	0.010 (0.000-0.016)	0.030 (0.006-0.050)	0.040 (0.037-0.080)	0.0003	0.0051	0.0002	0.1095
	TNF-α (single)	0.005 (0.000-0.021)	0.110 (0.070-0.230)	0.463 (0.276-1.010)	< 0.0001	< 0.0001	< 0.0001	0.0050
	IL-17 (single)	0.004 (0.000-0.020)	0.010 (0.000-0.030)	0.025 (0.006-0.162)	0.0734	0.1636	0.0313	0.3082
	IFN-γ/IL-2	0.000 (0.000-0.000)	0.004 (0.000-0.009)	0.004 (0.000-0.020)	§	-	-	-
	IFN-γ/TNF-α	0.000 (0.000-0.004)	0.040 (0.004-0.140)	0.258 (0.217-0.800)	< 0.0001	< 0.0001	< 0.0001	0.0035
	IFN-γ/IL-17	0.000 (0.000-0.000)	0.000 (0.000-0.000)	0.000 (0.000-0.013)	§	-	-	-
	IL-2/TNF-α	0.002 (0.000-0.005)	0.024 (0.010-0.042)	0.057 (0.029-0.217)	< 0.0001	< 0.0001	< 0.0001	0.2126
	IL-2/IL-17	0.000 (0.000-0.004)	0.000 (0.000-0.009)	0.027 (0.008-0.034)	0.0006	0.7456	0.0002	0.1870
	TNF-α/IL-17	0.000 (0.000-0.003)	0.000 (0.000-0.005)	0.020 (0.004-0.023)	0.0011	0.4198	< 0.0001	0.0065
	IFN-γ/IL-2/TNF-α	0.000 (0.000-0.001)	0.020 (0.009-0.120)	0.060 (0.027-0.427)	< 0.0001	< 0.0001	< 0.0001	0.0712
	IFN-γ/IL-2/IL-17	0.000 (0.000-0.000)	0.000 (0.000-0.000)	0.000 (0.000-0.000)	§	-	-	-
	IFN-γ/TNF-α/IL-17	0.000 (0.000-0.000)	0.000 (0.000-0.000)	0.000 (0.000-0.001)	§	-	-	-
	IL-2/TNF-α/IL-17	0.000 (0.000-0.002)	0.003 (0.000-0.018)	0.030 (0.002-0.035)	0.0008	0.0329	0.0024	0.0871
	IFN-γ/IL-2/TNF-α/IL-17	0.000 (0.000-0.000)	0.000 (0.000-0.000)	0.000 (0.000-0.002)	§	-	-	-
MTBk	IFN-γ (single)	0.007 (0.000-0.024)	0.285 (0.012-0.920)	0.593 (0.243-1.135)	< 0.0001	< 0.0001	< 0.0001	0.3114
	IL-2 (single)	0.022 (0.010-0.060)	0.087 (0.030-0.141)	0.130 (0.040-0.193)	0.0023	0.0012	0.0193	0.5590
	TNF-α (single)	0.026 (0.010-0.048)	0.090 (0.070-0.180)	0.243 (0.136-0.557)	< 0.0001	< 0.0001	< 0.0001	0.0354
	IL-17 (single)	0.056 (0.030-0.098)	0.120 (0.080-0.180)	0.510 (0.153-0.890)	< 0.0001	0.0033	0.0005	0.0175
	IFN-γ/IL-2	0.000 (0.000-0.000)	0.000 (0.000-0.002)	0.002 (0.000-0.005)	§	-	-	-
	IFN-γ/TNF-α	0.000 (0.000-0.002)	0.040 (0.009-0.120)	0.120 (0.054-0.370)	< 0.0001	< 0.0001	< 0.0001	0.1016
	IFN-γ/IL-17	0.000 (0.000-0.000)	0.000 (0.000-0.002)	0.000 (0.000-0.017)	§	-	-	-
	IL-2/TNF-α	0.006 (0.001-0.010)	0.020 (0.010-0.060)	0.038 (0.030-0.064)	0.0001	0.0030	0.0004	0.1599
	IL-2/IL-17	0.028 (0.008-0.051)	0.049 (0.023-0.110)	0.170 (0.069-0.317)	0.0072	0.0166	0.0073	0.0732
	TNF-α/IL-17	0.005 (0.001-0.019)	0.010 (0.003-0.020)	0.029 (0.011-0.062)	0.0695	0.3819	0.0091	0.0512
	IFN-γ/IL-2/TNF-α	0.000 (0.000-0.000)	0.003 (0.000-0.010)	0.008 (0.003-0.053)	§	-	-	-
	IFN-γ/IL-2/IL-17	0.000 (0.000-0.000)	0.000 (0.000-0.000)	0.001 (0.000-0.003)	§	-	-	-
	IFN-γ/TNF-α/IL-17	0.000 (0.000-0.000)	0.000 (0.000-0.001)	0.000 (0.000-0.005)	§	-	-	-
	IL-2/TNF-α/IL-17	0.008 (0.001-0.024)	0.030 (0.010-0.050)	0.080 (0.015-0.143)	0.0058	0.0088	0.0210	0.2424
	IFN-γ/IL-2/TNF-α/IL-17	0.000 (0.000-0.000)	0.000 (0.000-0.000)	0.001 (0.000-0.004)	§	-	-	-

§ Insufficient number of unique values to calculate valid p-value.
